# Entering First-in-Human Clinical Study With a Single-Strain Live Biotherapeutic Product: Input and Feedback Gained From the EMA and the FDA

**DOI:** 10.3389/fmed.2021.716266

**Published:** 2021-08-11

**Authors:** Jeanne-Céleste Paquet, Sandrine P. Claus, Magali Cordaillat-Simmons, Wilfrid Mazier, Georges Rawadi, Laure Rinaldi, Frédéric Elustondo, Alice Rouanet

**Affiliations:** ^1^Ysopia Bioscience, Bordeaux, France; ^2^Pharmabiotic Research Institute – PRI, Narbonne, France

**Keywords:** Microbiome, Live Biotherapeutic Products, regulatory science, Food and Drug Administration, European Medicines Agency, FIH clinical trial, LBP

## Abstract

During the last decade, a plethora of novel therapies containing live microorganisms as active substance(s) has emerged with the aim to treat, prevent, or cure diseases in human beings. Both the Food and Drug Administration (FDA) and the European Directorate for the Quality of Medicines and Health Care (EDQM) codified these biotherapies as Live Biotherapeutic Products (LBPs). While these innovative products offer healthcare opportunities, they also represent a challenge for developers who need to set the most suitable designs for non-clinical and clinical studies in order to demonstrate a positive benefit/risk ratio through relevant quality, safety, and efficacy data that are expected by the drug competent authorities. This article describes how YSOPIA Bioscience, supported by the Pharmabiotic Research Institute (PRI), addressed the regulatory challenges during the early development phase of their single-strain LBP, Xla1, in order to obtain the necessary authorizations to bring this drug to the clinical stage.

## Introduction

Effects of the microbiome on human health were described for the first time at the beginning of the 1900's by Elie Metchnikoff (1). Recently, improvement of the efficiency of sequencing methods has revived interest in the microbiome field and has enabled microbiologists to perform genomics analysis and break down complex ecosystems such as human fecal material (2, 3). From then on, numerous correlation and causality relationships between microbiome and pathologies have been uncovered (4, 5). The treatment of disease by way of microbiome intervention is now in the realm of possibility, and several microbiome-based therapies are currently in development for this purpose, including Live Biotherapeutic Products (LBPs).

Firstly defined by the Food and Drug Administration (FDA), LBPs are biological medicinal products containing live microorganism(s) as active substance(s) (6, 7) (AS). Despite the emergence of guidelines (6, 7), a number of gaps remain unaddressed to support the development of these new AS, in particular regarding how drug regulatory requirements should be addressed in practice. Indeed, LBPs face specific challenges inherent to their biological characteristics and modes of action (MoA), and as such, require special considerations for quality, safety and efficacy documentation before being used in humans. This is why the Pharmabiotic Research Institute (PRI) was created in 2010; in order to support its members in their efforts to develop and register microbiome-based medicinal products in the European Union (EU). As a non-profit entity, the PRI has developed a collaborative approach to identify and clarify the regulatory and scientific requirements that will be expected from the European competent drug authorities when market approval will be sought for these innovative therapies.

YSOPIA Bioscience is a French pharmaceutical biotechnology company developing microbiome-based therapies focused on keystone bacteria. YSOPIA's first drug development program aims to exploit the potential of *Christensenella minuta* DSM 33407 with its Drug Product (DP), Xla1, as a novel biotherapy to treat obesity and associated metabolic disorders.

As drug development is aiming at global markets, and in absence of international harmonization of the regulatory expectations for LBPs, YSOPIA, supported by the PRI, engaged in discussions with the EMA and the FDA to adapt its development strategy to their evaluation and comments. YSOPIA submitted a pre-Investigational New Drug (IND) package to the FDA (in 2019) and requested a scientific advice to the EMA (in 2020) based on two briefing packages containing the same level of information. Knowing this, the EMA requested the minutes of the pre-IND meeting with the FDA. The company's strategy to provide evidence of quality, safety and efficacy of its LBP candidate has therefore been fostered by feedbacks from both the EMA and the FDA, shedding further light on an area where the regulatory agencies are in need of relevant data and scientific rationale.

In the present article, the authors aimed at highlighting key regulatory concepts specific to LBPs that were raised by both competent authorities to support strategic decisions that must be made when designing comprehensive development plans for LBPs. Since the two competent authorities offer distinct procedures (i.e., pre-IND leading to clinical trial authorization for the FDA vs. scientific advice as a tool to engage in early discussions with the EMA) nuances have to be expected and will be highlighted. Moreover, for the sake of clarity, the article will be presented based on the Common Technical Document structure (8) relevant for both applications [i.e., Investigational New Drug (IND) and Investigational Medicinal Product (IMPD)] starting with manufacturing considerations, then addressing pre-clinical and clinical aspects.

## Considerations Required for LBP Quality Documentation

### Cell Banks Establishment and Management

LBP development begins with bacterial strain isolation, banking, and characterization. This step often involves several developmental steps of manipulation and culturing before the initial cell bank, the Research Cell Bank (RCB), can be finalized. Both in the EU and in the United States (U.S.), quality documentation must include the description of the strain's origin (material from which the strain was isolated) and strain's culture/passage history before finalization of the bank (6, 7). Furthermore, when strains are isolated from human biological material, information on the donor must be documented (9). However, the level of documentation required about the donor is not currently specified in any guidelines; therefore, developers must ensure that appropriate data are obtained at the time of collection, considering potential ethical limitations. Based on guidelines previously published for biologicals (9), the following information (Table 1) seemed appropriate to document the origin of a strain:

**Table 1:** Essential information to document the origin of the strain.

**Information to document**	**DATA to gather/collect**
Original source of cells from which the DS was derived	For example, fecal material
Donor(s) information	Relevant information potentially impacting the safety of the active substance such as for example age, sex, general physiological condition, state of health or medical history, body mass index, absence of pathogenic agents, absence of travel, and antimicrobial treatments during a relevant period before sampling.
Selection modalities and culture/passage history of the strain	Laboratory documentation and traceability

In the case of LBPs, the management of the cell banking system is key as it contains the AS of the product itself and, it may therefore directly influence the quality of the final product, as well as its safety and efficacy (10, 11). After comprehensive characterization of the strain(s), the Master Cell Banks (MCB) and Working Cell Banks (WCB) must be prepared in GMP environment from the RCB in order to answer regulatory requirements for the production of human therapies. MCB and WCB must be characterized exhaustively and their preparation process should be described in detail (9). A WCB may not always have to be generated prior to Phase 1 clinical study as it was acceptable for the FDA (6). However, both agencies advise preparing the WCBs as early as possible, pointing out that a WCB is an essential component of any acceptable quality development allowing to keep MCB as long as possible.

During our interactions with the EMA, their representatives pointed out that the rationale of the selection of the desired strain and its purity should also be confirmed with relevant data. Indeed, we noted that it was highly important that LBP developers provide a rationale for the isolation and selection of the strain to be banked. This means that integration of quality aspects as well as potential safety and efficacy features of the AS must be considered early on in the development plan.

### Comprehensive Strain Characterization

Characterization of the microbial cells used to establish cell banks is an essential part of LBP quality documentation as it describes the identity, potency, quality, and purity of the AS. Both the FDA and the European Directorate on Quality of Medicines and Health Care (EDQM) have published their expectations regarding the characterization of the microorganism used as AS in LBPs (6, 7). For strain characterization documentation, neither the EMA nor the FDA required any additional elements to those specified in the guidelines (6, 7).

Developers must provide identification of the microorganism at both species and strain levels and the FDA especially recommends using at least two complementary methods for this identification (Table 2). Furthermore, in the case of LBPs, strain(s) characterization is also part of the safety documentation.

**Table 2:** Quality requirements from both EU and US regulatory authorities for strain characterization.

**Required information**		**Selected tests and assays**
Genotyping	Identification at species level	16S rDNA genotyping
	Identification at strain level Antimicrobial resistance genes Virulence genes Presence of mobile genetic elements Plasmid detection Bacteriophage-related DNA insertions Transposons	Whole genome sequencing
Phenotyping	Identification at strain level	MALDI-TOF
	Morphology identification Gram staining Cell shape and size	Microscopy
	Growth characteristics	Growth kinetics, pH tolerance, aerotolerance, bile acid resistance
	Motility and sporulation	Wirtz-Conklin method
	Antibiotic sensitivity profile	Antibiogram along minimum inhibitory concentrations
	Enzymatic activity	API 20A anaerobic test, API rapid ID32A, API ZYM, oxidase, and catalase activity
	Bacterial endotoxins	*• Method A*: gel-clot technique (12)*• Method B:* turbidimetric technique (12)*• Method C:* chromogenic technique (12)

Microorganism characterization must include an assessment of antibiotic resistance through genotypic and phenotypic assessments (i.e., antibiograms). Developers should determine minimum inhibitory or minimum bactericidal concentrations to a selected panel of antibiotics identified beforehand based on a justified scientific rationale considering the nature of the strain (e.g., Gram staining) and the targeted population (13–15). Then, for any antibiotic resistance identified, it is required to determine whether this resistance is transferable from the microorganism to the targeted microbiota. Transfer of antibiotic resistance is not acceptable as it may represent a long-term risk for patients. Transferability of antibiotic resistance genes may be anticipated through genome analysis, if these genes are positioned on transposons, plasmids or any other mobile genetic elements, risk of transferability is present. As for antimicrobial resistance, the presence of virulence genes and their potential for transfer must also be addressed. The EMA has specified that the whole genome sequence of the strain must be included in the final product's dossier, as well as the detailed list of the identified antibiotic resistance genes, multidrug resistance clusters, putative virulence factor genes, and mobile genetic elements. However, there is to date no specific guidelines from the EMA or the FDA that provide details regarding the quality of the genome sequencing and associated bioinformatic analysis.

To rule out the risk of infection, it is also necessary to evaluate the translocation potential of the strain. With respect to the relationship between translocation potential and pathogenicity two aspects should be addressed: (1) the ability of the strain to cross the mucosal barrier, and, (2) the potential to induce a pathogenic reaction upon passage to the systemic circulation (inflammation, sepsis, or bacteria-mediated organ damage) (16). Therefore, a suitable assay should be developed to assess translocation potential, that should be aligned with the characteristics of the intended population.

The table below (Table 2) is a summary of the tests and assays proposed by YSOPIA to the EMA and the FDA in order to document a comprehensive characterization of the AS (*C. minuta* DSM 33407) in line with the aforementioned guidelines (6, 7).

### Large Scale Production of the Strain (Active Substance)

Culture is a critical step of the manufacturing process for LBPs; therefore, relevant in-process controls should be anticipated, and acceptance criteria should be established in order to minimize variability and to ensure safety of the process. The EMA strongly encourages applicants to establish in-process controls and acceptance criteria for critical steps of the manufacturing process of Phase 1 material. Moreover, large-scale culture of a microorganism requires a profound expertise and mastery of the strain intended for cultivation. Being a living organism, a bacterial strain has specific growth requirements; thus, the culture medium and environmental conditions have to be tightly controlled. Of note, strictly anaerobic bacteria such as *C. minuta* represent an additional challenge as they cannot be cultivated in presence of oxygen. Moreover, raw materials that will compose the culture medium must comply with Good Manufacturing Practices (GMP) (17).

The large-scale production of microorganisms which are intended to be kept alive in the final product raises additional challenges related to the reduction of risk of accidental cross-contamination. Microbiological examination and strain identification are therefore a critical part of the control strategy in order to ensure the quality and safety of LBPs. Microbiological quality examination includes aerobic microbial contamination count (AMCC), combined yeasts/molds contamination count (YMCC) and tests for specified micro-organisms such as 16S rDNA genotyping. Both the European and American pharmacopeia have described limits and methods specific to LBPs (7, 18, 19), or applicable to all non-sterile medicinal products (20–22) respectively. Besides, as mentioned during interactions with competent authorities, applicants have the responsibility to demonstrate the suitability of the selected methods as well as the viability of the tested microorganisms.

### Control Strategy of the Manufacturing Process

The novelty and complexity of the biological analytical techniques involved in the characterization and manufacturing control strategy of LBPs also represent a challenging aspect of quality documentation for developers.

Additional issues for LBP characterization, manufacturing process, and their compliance with global drug regulatory requirements are related to the analytical methods employed for these products. As for any other drug, regulatory agencies require the accurate description of analytical methods used during the drug manufacturing process, especially those deployed for the drug characterization and establishment of specifications, as they will consequently be applied to in-process controls and release tests. Furthermore, the EMA reminded us that suitability for use and validation of these analytical methods needs to be demonstrated and supported by data in accordance with international and regional guidance (23, 24).

Analytical methods used for LBPs principally include sequencing, plate count and cell count. For LBPs, the strains' genotypes often guide lead candidate selection and, when a specific strain is selected as a drug candidate, its genome acts as its “*official passport”* and will be the basis of genotypic controls for identity all along the drug quality control process. While plate count and cell count methods are generally exploited for purity and potency determination, several challenges reside in the robust execution and establishment of these methods including the execution of such methods under GMP conditions and in routine production. Furthermore, establishing a potency assay for a LBP may be challenging since the exact MoA is not always completely deciphered, rendering difficult the identification and validation of suitable tests for potency control. Finally, in the case of anaerobic strains such as *C. minuta*, an additional challenge resides in the execution and establishment of such assays under anaerobic conditions.

### Batch to Batch Consistency and Stability

The final major industrial challenge of live ASs resides in ensuring batch-to-batch consistency of the Drug Substance (DS) and DP. Indeed, variations in the quantity of live microorganisms between batches is greater than what would be expected for other types of drugs. This is addressed by broadening product specifications (for both DS and DP) in terms of viable cell levels and/or Colony Forming Units (CFU) per grams/liters.

Furthermore, the amount of AS within the final products is subject to higher instability than other types of drugs. This can lead to a large variance in viable cell levels and CFUs between batch release, which impacts on the end of shelf life. Neither of the agencies had issues with this principle, as long as appropriate stability data were provided.

In summary, quality risk management principles are crucial considering the inherent variability of biological materials and should be respected or adapted to develop the control strategy of the manufacturing process in order to optimize, as much as possible, the consistency of LBP production (25).

## Considerations Required for LBP Non-Clinical Documentation

The objectives of the non-clinical safety studies are to assess pharmacological and toxicological effects prior to initiation of human studies and throughout clinical development (26). Before a Phase 1 clinical trial, a preclinical program should cover the information needed for a safe transposition of the drug from animals to humans.

The following section will present the non-clinical program developed by YSOPIA and presented to the EMA and the FDA. The non-clinical plan (Table 3) is simplified in comparison to “conventional” non-clinical package for several reasons:

- The bacteria used as AS of the LBP (*C. minuta* DSM 33407) is a commensal bacterium isolated from a healthy human and has already been reported to be linked to a positive clinical outcome (27),
- The effects of a strain on the human gastrointestinal (GI) tract cannot be accurately mimicked in any animal model currently available (28, 29)
- In order to reproduce as accurately as possible the human microbiome ecology, the non-clinical simulation of the effects of the AS (*C. minuta* DSM 33407) was conducted using the *ex vivo* GI SHIME® model (30, 31),
- DP's effect on the ecology of the microbiome will be more deeply evaluated during the clinical trials which will be conducted directly on the target population.

**Table 3:** Non-clinical package proposed for pre-IND/SA.

	**Study conducted**	**Information collected**
Pharmacology studies	Model selection	Validate the most appropriate model to evaluate the impact of chronic treatment in future *in vivo* studies
	Strain selection	Identify a *C. minuta* candidate strain for further development as an LBP to target obesity
	*In vivo* efficacy	Address the efficacy of the drug candidate
	Dose ranging	Identify a putative dose-dependent efficacy of Xla1
Safety studies	Translocation	Demonstrate that Xla1 does not present any risk of treatment-induced bacterial infection
	High-dose tolerance and wash out	Evaluate the impact of an acute exposure to the highest concentration technically achievable

### Traditional Pharmacology, Pharmacokinetic, and Toxicology Studies

#### Pharmacology Studies

For LBPs, the pharmacological effects vary depending on the specific properties of the microorganism used as AS, every species or even strain having a unique biology. As in any drug development process, both the EMA and FDA ask developers to select and design preclinical studies according to the specific features of their drug candidate and in alignment with the target clinical condition. However, LBPs developers face challenges to meet these expectations because there are no standardized models where host-microbiome interactions can be accurately simulated, particularly in the context of metabolic pathologies (32).

However, the EMA expects demonstrations of causality between product administration and improvement of physio-pathological parameters. The preferred way to demonstrate a causal relationship is to establish a MoA. Indeed, as stated by the EMA, understanding how a drug works before it is tested in clinical trials is important. This facilitates drug monitoring on the target pathway in the patient. In addition, knowing how a medicine works may help predict and prevent adverse effects, and can also aid in the establishment of contingency plans in the event of unintentional harm to patients.

As previously explained (see Introduction & Control strategy of the manufacturing process), defining a clear MoA is not simple for LBPs as they usually act via multiple simultaneous pathways which can be directly mediated by interactions with the immune system, or indirect through gut microbiome modulation and production of active metabolites (33, 34). Once again, obtaining an exhaustive characterization of the MoA is complicated by the entanglement of the relationship between the microbiome and its host since reproducing this complex interaction in non-clinical models is very challenging (28, 29). The use of complex dynamic artificial models of organs (e.g., SHIME® model mentioned above) can be helpful to study physiochemical, enzymatic and microbial parameters in a controlled *in vitro* setting. It is therefore recommended to multiply complementary models to improve understanding of the various aspects of MoAs.

#### Dosing Rationale and Pharmacokinetic Assessment

As stated in the ICH S6(R1) guideline (26), it is difficult to establish a uniform way for pharmacokinetic (PK) studies for biotechnology-derived pharmaceuticals. Indeed, in the case of LBPs, the AS is not expected to penetrate the systemic circulation and reach distant organs. Therefore, YSOPIA did not carry out traditional PK studies and instead employed relevant *in vitro* biodistribution and host-microbiome interaction studies. Traditional dose ranging studies were performed in order to determine whether there was a dose-dependent relationship with the product efficacy. However, we did not observe any dose-effect relationship for our strain (cf. Dosing rationale & Pharmacokinetic assessment). As a consequence, both the EMA and the FDA acknowledged that traditional PK and toxicokinetic (TK) studies were not relevant for LBPs; however, it is recommended that developers should demonstrate through relevant studies (i.e., translocation studies) that the strain does not become systemically available.

There are currently no specific guidelines acknowledging a common approach to determine the dose for an LBP. The FDA proposes in the guideline “*Estimating the Maximum Safe Starting Dose in Initial Clinical Trials for Therapeutics in Adult Healthy Volunteers”* (35) several approaches to convert doses studied in animal models to humans for clinical trials. However, these conversion indices are based on standard DPs and do not apply to the specificity of live microorganisms and their inherent capability to reproduce within the host. Also, the generic method takes into consideration the body distribution and does not consider the restricted compartmentalization of an LBP in the GI tract. Furthermore, the EMA has stated in recent guidelines that it is upon the developer to identify and mitigate risks for first-in-human (FIH) clinical trials which include applying a scientific rationale in the selection of the starting dose (36). Therefore, LBP developers should propose an alternative and suitable approach to estimate a LBPs' human equivalent dose. Here is a list of questions that was raised by the EMA on this subject:

- *What part of the targeted organ should be covered with the microorganism to achieve the expected effect?*
- *Will the microorganism actually get there?*
- *How many microorganisms are needed to provide sufficient cover?*
- *Is the microorganism expected to grow and multiply at the site of action? How this could be monitor?*
- *How long does it take for the complete elimination of the microorganisms after administration has ceased?*

Based on the answers, developers acquire a body of knowledge which will serve as the basis of the dosage and administration schedules for their FIH studies.

### Toxicology Studies/Safety Assessment

The risk of transferability of antibiotic resistance to other bacteria and the risk of causing infection are the two risks commonly identified for LBPs. As such, drug competent authorities require developers to assess them as early as in the characterization studies (6). Consequently, data gathered through these studies will also be part of any safety documentation; notably, translocation studies.

These two risks (i.e., transfer of antibiotic resistance and infection) may also be associated with other risks specific to the species, to the strains and to the patient who will receive the treatment. In order to carry on a risk analysis, it is important to document any beneficial and/or adverse effect ever documented for the species and if available, for the strain to be used as AS. Then, assessment and documentation of all identified risks must be considered to design a relevant preclinical program that will allow to prepare an appropriate risk management plan for further clinical trials.

#### Translocation

As the DS of LBPs contains live bacteria, as mentioned above, a translocation assessment study is essential to demonstrate the absence of bacteria transfer into the systemic circulation in order to exclude any risk of treatment-induced bacterial infection for patients. To do so, relevant tissues must be analyzed using targeted qPCR along bacterial culture to detect potential live bacteria and prove that bacteria do not translocate. Bacterial translocation can be exacerbated by at least 3 mechanisms: altered intestinal barrier function, dysbiosis, and impaired host defense (37). Thus, the pathophysiology of the target population must be well-understood in order to adapt the translocation model.

#### Traditional Toxicology Studies

The understanding of the complex molecular pathways involved in the interaction between the microbiota and its host (human or animal) is only in its infancy. In accordance with recent guidelines applicable to biotechnological medicinal products (26, 36), a list of inherent risks associated with LBPs was identified and was addressed through relevant studies.

The FDA has considered that this package is appropriate to support the safety of Xla1 for a FIH study and that no further toxicity studies were needed at this stage of development. However, they specify that, if a safety signal arose during clinical development, the regulatory authority may request additional toxicity studies to address them.

It is more difficult to draw such definite conclusions from the feedback received from the EMA. The EMA anticipated that for a marketing authorization, the need for additional toxicity studies would depend on several factors. For example, if the AS is derived from healthy human commensal bacteria that are ingested in amounts within physiological range, and if it does not become systemically available, no additional traditional toxicity studied may be necessary. Otherwise, the safety of the bacteria will have to be substantiated. The EMA did not rule out that this could be done based on existing literature.

### GLP Compliance

Most non-clinical studies are generally conducted in agreement with the Good Laboratory Practice (GLP) certification. As a result, this usually leads developers to subcontract their studies to GLP-certified Contract Research Organizations (CROs). However, handling strictly anaerobic bacteria, like *C. minuta*, requires specific study conditions, which is an important limitation for many specialized CROs that are unable to perform anaerobic GLP microbiology in their facilities. With this in mind, regulatory authorities were receptive to these difficulties and accepted that non-clinical programs may be performed in non-GLP facilities that can demonstrate an adequate level of quality. Yet, the EMA highlighted that potential aspects of the non-clinical studies that would deviate from standard GLP conditions should be discussed.

## Considerations Required for LBP Clinical Documentation

Besides the usual clinical challenges posed by every targeted indication and associated pathophysiological mechanisms, LBP development has its own specific set of challenges to enter FIH clinical trial. Indeed, as mentioned above, because of poor translation from animal models to humans, non-clinical studies for LBPs provide limited information in comparison to the level of predictability obtained from non-clinical programs designed for other types of drugs. Consequently, LBP developers need to take into account this high level of uncertainty when designing their FIH clinical trials.

For Xla1 FIH (38) (NCT04663139), Phase 1 clinical trial was designed in order to test a daily oral single dose, and to evaluate safety, tolerability and impact on gut microbiota following introduction of Xla1 in two subsequent parts:

- Part 1: An open phase in normal weight healthy volunteers (HV) receiving Xla1,
- Part 2: A randomized, parallel, double-blind, placebo-controlled phase in overweight or obese adult patients (Stage 1) receiving either Xla1 or placebo.

### Study Design and Safety Plan

Like every DP, the development of an LBP presents challenges regarding the design of clinical trials that need to conform to current guidelines or scientific recommendations adapted to the assessment of safety and efficacy in the target population (39, 40). However, LBPs bear also specific challenges related to the living nature of the AS. Inclusion and exclusion criteria should always be defined based on specific risks identified for the target population. Special attention should be given to risks commonly accepted for LBPs (i.e., transferability of antibiotic resistance to other commensal microbes and translocation into the systemic circulation) in addition to the specific risks identified during the early development steps.

**Figure 1:**
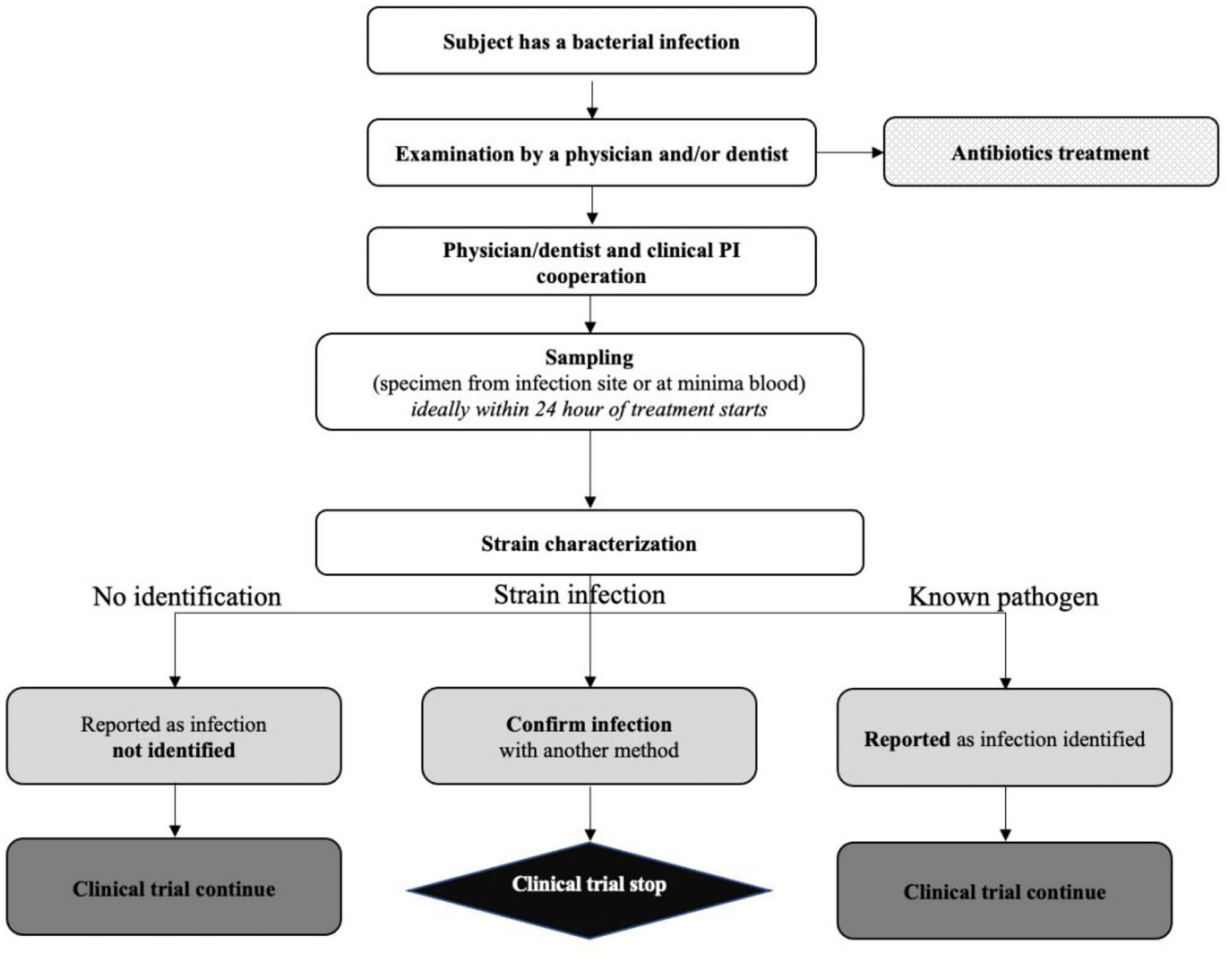
YSOPIA risk management plan to manage potential clinical infection applied during phase 1 trial.

Both the FDA and the EMA were concerned about the risk of causing a systemic infection through administration of live bacteria. Therefore, a detailed management procedure had to be provided (Figure 1), including a description of antibiotic therapies that have proven efficacy against the DS, both through intravenous and oral administration.

### Refinement of Dosing Regimen

The purpose of the FIH clinical study is to assess the product's tolerability. In general, for products where toxicity is directly related to the dose, the dose range covered in Phase 1 should be larger than the dose range applied in later trials. However, for Xla1 a dose-effect relationship was not observed in non-clinical studies (cf. Dosing rationale & Pharmacokinetic assessment), the risks identified were consequently not considered as related to the dose and a dose-escalation scheme in Xla1 Phase 1 study design was not considered as relevant.

The choice of testing solely a single dose was not challenged by the FDA, but the EMA did raise some questions. The EMA considered that the risk of infections may be higher when using a high dose of microorganisms, and therefore would recommend evaluating a lower dose to minimize the risk of translocation. The EMA recommended that the trial should ideally include a wide range of doses, starting with the minimal dose without any effect and ending with the highest possible dose, considering safety, efficacy and practical considerations (i.e., number of capsules or volume to be ingested). It seems that EMA would consider insufficient to only assess the dosing schedule (e.g., single-dose, repeated administration, or multiple dose single administration). The FDA indicated the need for multiple dose assessment during later phases of the drug development program.

The EMA also pointed out the fact that sponsors must consider that if some of the participants of the clinical trial are healthy, they might already be carriers of the commensal bacteria and then could react differently to the administration in comparison to diseased patients who are assumed to be low carriers. Indeed, this is particularly relevant to our LBP that is based on a low abundant strain of *C. minuta*. Thus, healthy volunteers might be exposed to an unusual high dose of *C. minuta* after the administration of Xla1 and such overdosage may potentially lead to unexpected adverse events. In order to evaluate and address such risk, thoroughly monitoring of the microbiome of all participants in order to collect enough longitudinal data was proposed. Analysis of these data will enable to accurately evaluate modifications of the microbiome composition over the course of the study. In addition, 4-week wash-out period to monitor and assess the engraftment of the strain was included. No follow-up long-term assessment was proposed. Neither the EMA nor the FDA did require a long-term evaluation of the study and both accepted this proposal.

## Discussion

Submitting the same briefing package through the EMA and the FDA regulatory process has given us some insights into the mindset and perspective of both regulatory agencies. Although the EMA had knowledge of the feedback received from the FDA, their response to the approach in addressing regulatory challenges for LBPs was different. It is important to point out that while the responses provided by the FDA are decisive and binding for entry into the Phase 1 clinical trial, this is not within the remit of the EMA at this stage. Indeed, while the pre-IND procedure allows the FDA to authorize clinical trials for drug products, scientific advices from the EMA are offered with the objective of de-risking development and exchanging on key issues before clinical trial authorizations are submitted nationally. Such procedural difference may explain the differing responses from the two agencies, which were, nevertheless, aligned on many aspects and rather complementary on others. A clear distinction in the philosophy of the two agencies regarding LBPs may however be pointed out.

The FDA provided with straight answers on the early development of the biotherapy, while the feedback received from consultation at the EMA covered the long-term vision of the drug development. In both situations, the authorities were highly concerned about patient safety, but both were supportive and open to the proposal of an innovative non-clinical package that they considered appropriate to the specific nature of LBPs. To this regard, the two authorities were true to their longstanding goal of supporting innovative medical care even if they have to juggle between benefits and risks that unconventional medicinal products may represent without one being at the expense of the other.

## Conclusion

In August 2020, IND authorization was granted to Xla1 allowing Phase 1 clinical trial to begin. The strategy developed to address the regulatory challenges of a single-strain LBP may therefore be considered as successful, or at least, as relevant for the FDA.

This experience demonstrates that when guidelines do not exist for a specific type of product, interactions with competent authorities through scientific advice or pre-IND meetings are key to resolve uncertainties and de-risk developments of innovative products. Furthermore, this approach enables regulators to better understand innovative biotherapies and their associated challenges, allowing them to better define areas where specific guidances are needed. For all these reasons, it is important to engage with competent regulatory authorities at an early stage in order to drive a comprehensive and successful development when dealing with an innovative therapy.

## Author Contributions

JCP, FE, and AR planned, outlined and coordinated the work of co-authors, and they drafted and edited the manuscript. JCP, WM, LR, and FE proceeded to the implementation of the CMC and non-clinical and clinical strategy of Yso1 described in this article. JCP submitted the manuscript. SC, MC-S, WM, GR, and LR edited and proceed to critical revision of the manuscript. All authors contributed to the article and approved the submitted version.

## Conflict of Interest

JCP, SC, WM, GR, LR, and FE are employees of YSOPIA Bioscience. MC-S and AR are employees of the Pharmabiotic Research Institute.

## Publisher's Note

All claims expressed in this article are solely those of the authors and do not necessarily represent those of their affiliated organizations, or those of the publisher, the editors and the reviewers. Any product that may be evaluated in this article, or claim that may be made by its manufacturer, is not guaranteed or endorsed by the publisher.
